# Clostridioides difficile Phosphoproteomics Shows an Expansion of Phosphorylated Proteins in Stationary Growth Phase

**DOI:** 10.1128/msphere.00911-21

**Published:** 2022-01-05

**Authors:** Wiep Klaas Smits, Yassene Mohammed, Arnoud H. de Ru, Valentina Cordo', Annemieke H. Friggen, Peter A. van Veelen, Paul J. Hensbergen

**Affiliations:** a Department of Medical Microbiology, Leiden University Medical Center, Leiden, The Netherlands; b Center for Proteomics and Metabolomics, Leiden University Medical Center, Leiden, The Netherlands; c Proteomics Centre, University of Victoria, Victoria, BC, Canada; University of Iowa

**Keywords:** *C. difficile*, growth phase, phosphorylation, posttranslational modification, proteomics

## Abstract

Phosphorylation is a posttranslational modification that can affect both housekeeping functions and virulence characteristics in bacterial pathogens. In the Gram-positive enteropathogen Clostridioides difficile, the extent and nature of phosphorylation events are poorly characterized, though a protein kinase mutant strain demonstrates pleiotropic phenotypes. Here, we used an immobilized metal affinity chromatography strategy to characterize serine, threonine, and tyrosine phosphorylation in C. difficile. We find limited protein phosphorylation in the exponential growth phase but a sharp increase in the number of phosphopeptides after the onset of the stationary growth phase. Our approach identifies expected targets and phosphorylation sites among the more than 1,500 phosphosites, including the protein kinase PrkC, the anti-sigma-F factor antagonist (SpoIIAA), the anti-sigma-B factor antagonist (RsbV), and HPr kinase/phosphorylase (HprK). Analysis of high-confidence phosphosites shows that phosphorylation on serine residues is most common, followed by threonine and tyrosine phosphorylation. This work forms the basis for a further investigation into the contributions of individual kinases to the overall phosphoproteome of C. difficile and the role of phosphorylation in C. difficile physiology and pathogenesis.

**IMPORTANCE** In this paper, we present a comprehensive analysis of protein phosphorylation in the Gram-positive enteropathogen Clostridioides difficile. To date, only limited evidence on the role of phosphorylation in the regulation of this organism has been published; the current study is expected to form the basis for research on this posttranslational modification in C. difficile.

## INTRODUCTION

Proteins in organisms from all domains of life can be functionally altered by posttranslational modifications (PTMs) that are often species dependent (1). PTMs in bacteria generally occur at much lower levels than in eukaryotes (2). PTMs serve to regulate activity in response to environmental conditions, as they generally occur faster than transcription-translation (2); they can also be used to tune phenotypic diversity (3). Among the high variety of PTMs (1), protein phosphorylation is one of the best-studied examples, partially attributable to major developments in phosphoproteomics.

To date, phosphorylation has been identified on the side chains of different amino acids (aa): serine (Ser), threonine (Thr), tyrosine (Tyr), histidine (His), arginine (Arg), lysine (Lys), aspartate (Asp), and cysteine (Cys) (1). The chemistries involved in the phosphorylation of different amino acids are different. Phosphorylation on Ser/Thr/Tyr results in more stable phosphoester bonds, whereas His/Lys/Arg phosphorylation results in thermodynamically unstable phosphoamidates (1). Phosphorylation on Asp leads to a mixed phosphoacylanhydride, and modification of cysteine, finally, leads to a phosphothiolate (1). Ser/Thr/Tyr phosphorylation appears to be most common in bacteria (4), but this may relate to their thermodynamic stability.

In canonical protein phosphorylation, a phosphate group is transferred to a protein through the action of a kinase and can be removed by a phosphatase; sometimes the kinase and phosphatase functions are encoded by the same bifunctional enzyme (1). Often, the response of bacteria to environmental stimuli is dependent on so-called two-component systems that consist of a membrane-associated two-component sensor histidine kinase (HK) and a cytosolic response regulator (RR). When triggered, autophosphorylation of the HK on a histidine residue results in the transfer of the phosphate group to an aspartate residue on the response regulator. Subsequently, the phosphorylated RR can bind to DNA to regulate the transcription of target genes (1). Other His-phosphorylated proteins include the phosphoenolpyruvate-dependent sugar phosphotransferase system (PTS) proteins, with the exception of the EIIB component, which can be phosphorylated on Cys residues (5). As the name suggests, the donor for the phosphorylation of PTSs is not a kinase but phosphoenol pyruvate (PEP). The PTS phosphorylation cascade includes an unusual bifunctional kinase/phosphatase, called HPr, that in its *S*-phosphorylated form can interact with the transcription factor CcpA to regulate metabolic activity (5).

Phosphorylation on Ser/Thr is mediated by eukaryote-type Ser/Thr kinases (eSTKs), also known as Hanks-type kinases (6). Interestingly, there is some evidence that eSTKs may outnumber two-component systems (7). Phosphorylation on Tyr in bacteria is mediated by a unique family of proteins known as BY kinases (2, 8, 9).

The exploration of bacterial phosphorylation has greatly been stimulated by the development of mass spectrometry (MS)-based phosphoproteomics techniques that provide a snapshot of the site-resolved phosphorylation state of proteins under given conditions (10, 11). These techniques rely on strategies to enrich for phosphorylated peptides through, for instance, anti-phospho antibodies, strong cation exchange chromatography, chemical modifications, or immobilized metal affinity chromatography (IMAC) (2, 4). Overall, the picture that emerges from these experiments is that both the numbers of phosphorylation sites and the relative abundances of different types of phosphorylation differ between organisms (2, 4, 11, 12). Additionally, it was found that one protein can be dynamically phosphorylated on multiple different sites (4, 13).

In bacteria, phosphorylation has been implicated in diverse processes, such as cell cycle regulation, cellular differentiation, morphogenesis, metabolism, persistence, and virulence (1). Whereas phosphoproteomics initially focused on model organisms, such as the Gram-negative bacterium Escherichia coli (14) and Gram-positive bacterium Bacillus subtilis (15), phosphoproteomes have been determined for a large number of pathogens (16, 17). Though some of the effects on pathogenicity can be related to pleiotropic effects on cellular integrity, for instance, phosphorylation is also directly implicated in virulence by modulating virulence gene expression or factors involved in host establishment, as well as affecting the host immune system (10, 16). Finally, phosphorylation has also been found to affect antimicrobial resistance in multiple pathogens (16, 18). Together, these findings suggest that targeting phosphorylation might be a viable strategy for novel antimicrobial or antivirulence strategies (16, 18).

Despite the wealth of phosphoproteomic studies (16, 17), phosphorylation in clostridia is largely unexplored. A single study has been performed with Clostridium acetobutylicum, a solventogenic species with biotechnological relevance, and it identified a total of 61 phosphorylated proteins (19). To our knowledge, no studies have been carried out in pathogenic species such as Clostridioides difficile.

C. difficile is a major cause of health care-associated diarrhea and can cause a potentially fatal disease (C. difficile infection, or CDI) as a result of the production of toxins that affect the integrity of the colon epithelium (20–22). The development of CDI is clearly linked to a reduced diversity of the microbiota of the host, and consequently, restoring this diversity, for instance by fecal microbiota transplantations, has proven to be an effective treatment (23, 24). The transmission and persistence of C. difficile are dependent on its ability to form endospores, as these processes are severely inhibited in a mutant unable to form spores (25). Sporulation and many of the processes described in the preceding paragraph are subject to complex regulation. Based on homology with the Gram-positive bacterium B. subtilis, phosphorylation of key proteins likely regulates these processes (26–30). However, despite this homology, important differences in these regulatory pathways exist between B. subtilis and C. difficile (31, 32). Importantly, genetic investigations have revealed that the eSTK protein PrkC of C. difficile affects cell wall homeostasis and resistance to antimicrobials, though the molecular mechanisms through which this occurs have not been elucidated (33). In contrast, it was recently shown that PrkC directly affects cell division through phosphorylation of the peptidoglycan hydrolase CwlA (34). To date, this is the only reported substrate of the PrkC kinase.

Here, we report the first phosphoproteomic analysis of C. difficile based on an IMAC-based enrichment strategy. We show that phosphorylation is growth phase dependent and abundant in stationary growth phase and that several aspects of phosphorylation-dependent regulation appear to be conserved. Our results contribute to our understanding of phosphorylation in pathogens and pave the way for functional dissection of the role of kinases and phosphatases in C. difficile.

## RESULTS

### A one-step IMAC procedure allows the identification of phosphopeptides from C. difficile.

The number of phosphorylated proteins in bacteria is generally much lower than in eukaryotic cells (1). To benchmark our IMAC workflow, we therefore first analyzed a human cell line (JY cells) where we were expecting a relatively high number of phosphopeptides compared to the number in C. difficile. From three biological replicates, we were able to identify a total of 10,620 unique phosphopeptides with a good overlap between the different samples (see Fig. S2 in the supplemental material). These results are in line with those of other recent studies (35, 36) and show that our approach allows the reliable identification of phosphopeptides in complex samples.

10.1128/msphere.00911-21.2 FIG S2 Summary of the total number of phosphopeptides retrieved from JY cells. Overlap between the three biological replicates is represented by a Venn diagram. Download FIG S2, PDF file, 0.1 MB. Copyright © 2022 Smits et al. 2022 Smits et al. https://creativecommons.org/licenses/by/4.0/ This content is distributed under the terms of the Creative Commons Attribution 4.0 International license.

**FIG 1:**
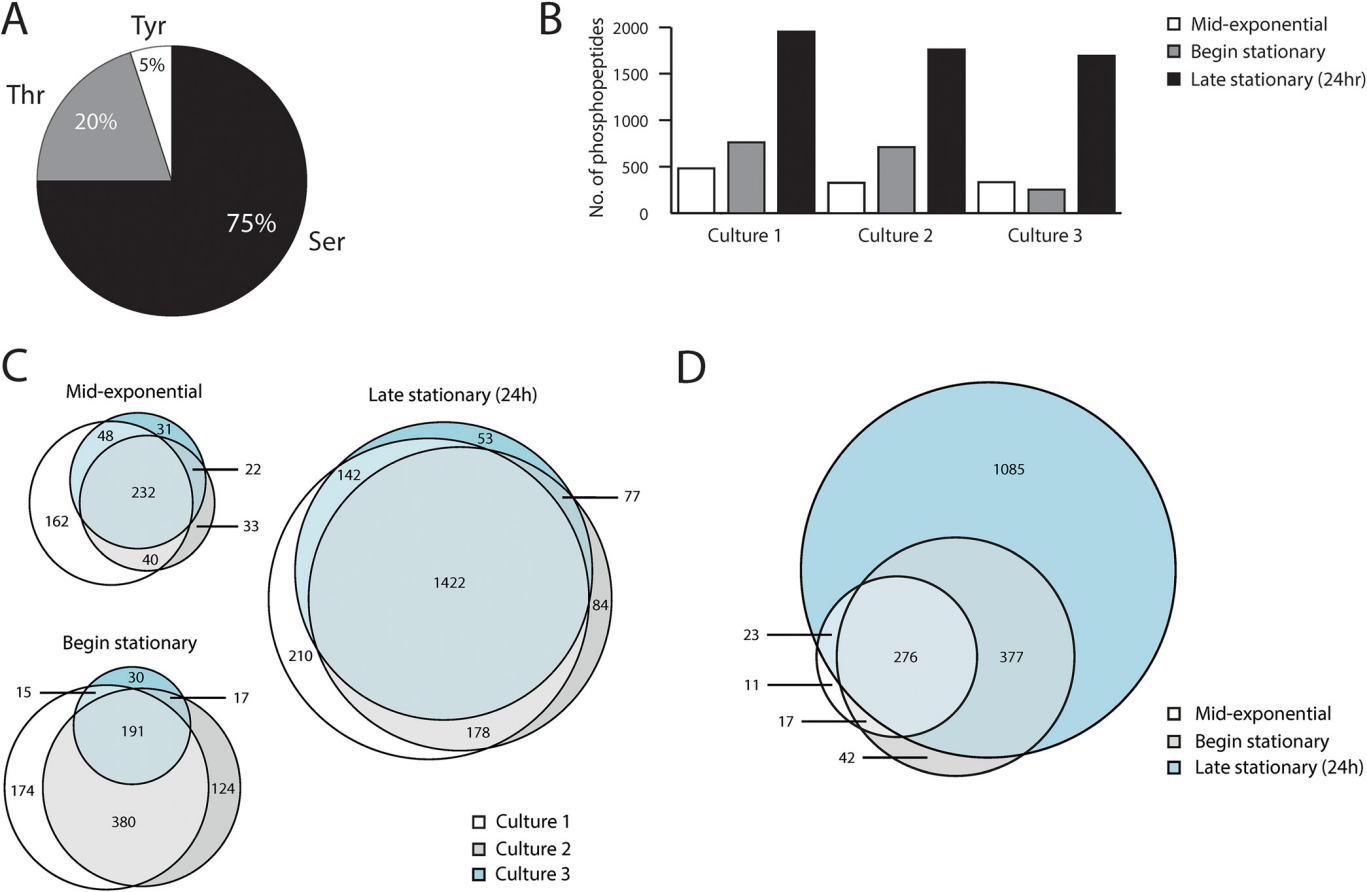
Global overview of phosphoproteins in C. difficile. (A) Pie chart indicating the percentages of Ser, Thr, and Tyr phosphorylation. (B) Numbers of identified phosphopeptides over time for the three biological replicates analyzed. For the definition of time points, see Fig. S1 in the supplemental material. (C) Concordance between the phosphopeptide identifications between the three biological replicates. (D) Changes in the identified phosphoproteome over time. Overlap in the identified phosphopeptides per time point for culture 2 is indicated in a Venn diagram.

Next, we analyzed the phosphoproteome of C. difficile at three different time points (mid-exponential phase, the start of stationary phase, and late stationary phase [24 h]) from three independently grown cultures. Overall, this resulted in the identification of almost 3,000 phosphopeptides (Table S1). Of these, 1,759 had a high localization probability (LP; above 0.95) for the correct assignment of the phosphorylation site. These sites were found on 1,604 unique tryptic peptides derived from more than 700 proteins. Within this set of peptides, 75% were found to be phosphorylated on a serine, 20% on a threonine, and 5% on a tyrosine residue (Fig. 1A). Based on these results, we conclude that using our experimental approach serine phosphorylation is most common in C. difficile, as also observed for other species (4, 11).

10.1128/msphere.00911-21.5 TABLE S1 Full data set of the phosphoproteome analysis and site assignment for Ser, Thr, and Tyr phosphorylation in C. difficile. Download Table S1, XLSX file, 0.5 MB. Copyright © 2022 Smits et al. 2022 Smits et al. https://creativecommons.org/licenses/by/4.0/ This content is distributed under the terms of the Creative Commons Attribution 4.0 International license.

10.1128/msphere.00911-21.1 FIG S1 Overview of growth and sampling of the C. difficile cultures. (A) Growth curve indicating obtained optical densities at 600 nm and sampling time points for this study. (B) Table summarizing protein yield from 100 mL (time point 1) and 50 mL (time points 2 and 3) of sample. Download FIG S1, PDF file, 0.4 MB. Copyright © 2022 Smits et al. 2022 Smits et al. https://creativecommons.org/licenses/by/4.0/ This content is distributed under the terms of the Creative Commons Attribution 4.0 International license.

To compare the different samples, we selected unique peptide sequences that were found to be phosphorylated, without considering phospho-isomers (i.e., phosphorylation at different sites in the same peptide). In general, we observed a progressive increase in protein phosphorylation during growth. We identified most phosphopeptides at later growth phases, with the most prominent increase between the samples taken at the onset of stationary growth phase and the samples from the 24-h cultures (Fig. 1B).

We assessed the reproducibility of the identifications between the different samples at the various time points. At the mid-exponential growth phase, 232 phosphopeptides were identified in all three samples, corresponding to 41% of the total number of peptides identified at this time point (Fig. 1C). For the second time point, 191 phosphopeptides were found in all three samples (20% of the total number identified at this time point). This relatively low percentage was due mainly to one of the samples, in which we found a lower number of phosphopeptides (Fig. 1B, culture 3), as the overlap between phosphopeptides was much higher between the other two cultures at this time point (63% of the total number). Of note, this was not due to technical variation, as analysis of a second, independently processed, sample from the same culture at the same time point showed similar low numbers of phosphopeptides. After 24 h, 1,422 phosphopeptides were common to all three samples (66% of the total number identified at this time point). Overall, this shows significant congruence between our phosphopeptide identifications, despite individual processing of all three biological replicates and time points.

Within one culture, most phosphopeptides observed at the earlier time points were also observed at later stages (as exemplified for culture 2 in Fig. 1D), suggesting a gradual increase in the repertoire of phosphoproteins rather than a large-scale reprogramming. This is also clear from the fact that 155 phosphopeptides were found in all samples at all time points. Nevertheless, we also observed a limited set of proteins that appear to be phosphorylated predominantly, if not exclusively, in the exponential growth phase (Table S1) and are absent from late-stationary-growth-phase samples. One such example is discussed further below.

**FIG 2:**
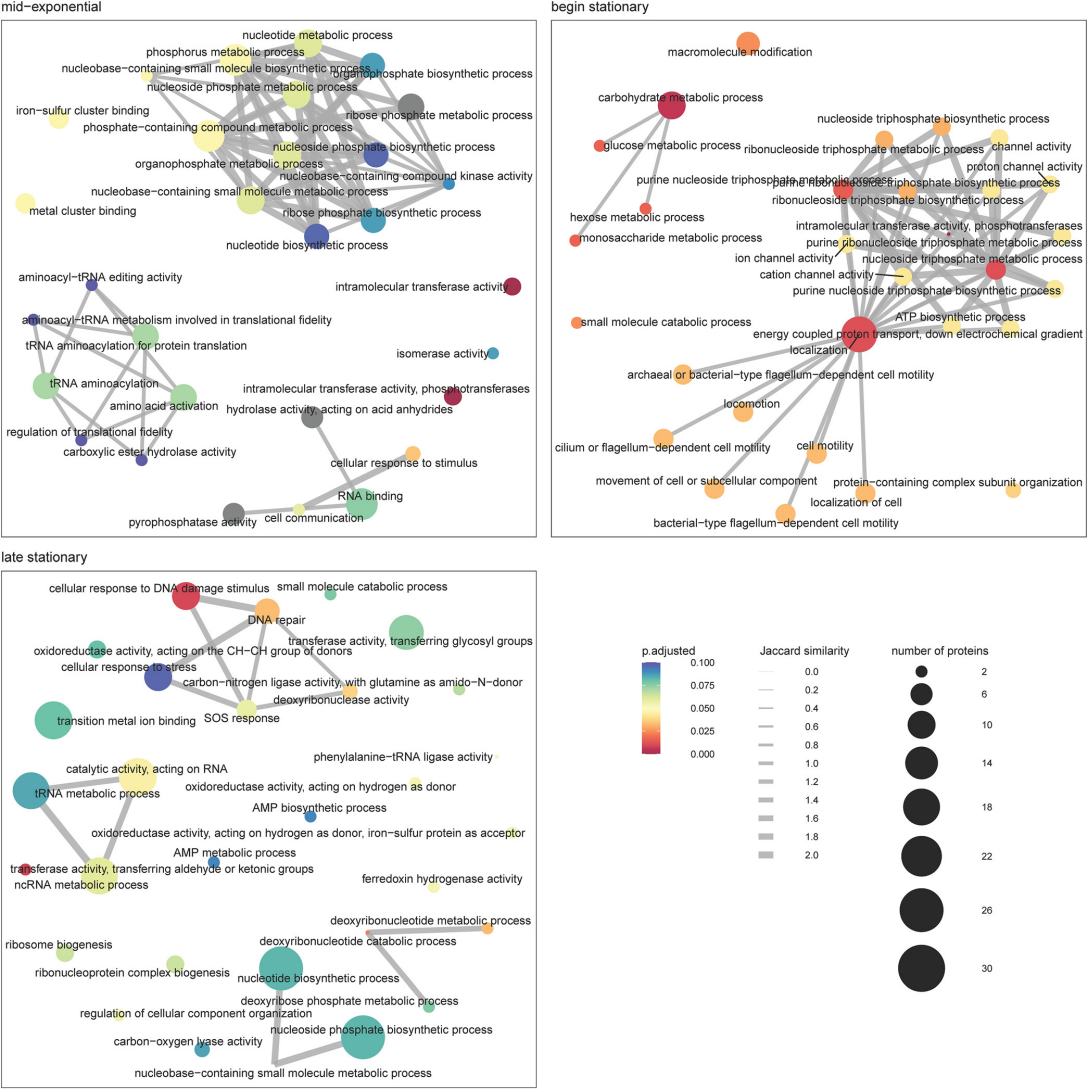
Enrichment maps of gene ontology terms obtained from C. difficile phosphoprotein set enrichment analysis. Three enrichment maps correspond to the three time points analyzed in this study (mid-exponential, beginning stationary, and late stationary growth phases); each map is limited to the top 30 enriched terms. The leaves represent gene ontology terms, and the edges correspond to the Jaccard similarity between the leaves based on the shared proteins.

We performed protein/gene set enrichment analysis of the phosphorylated proteins identified and determined the molecular functions and biological processes associated with these proteins. Figure 2 represents the enrichment maps for each of the three time points, providing an overview by aggregating the enriched terms according to the shared protein set. Comparing the enrichment maps side by side revealed that during the first two stages, there were concentrated, interconnected clusters of functions/processes, i.e., performed by the same set of proteins, a trend that was lost in the late stationary growth phase. At the mid-exponential growth phase, these clusters were largely associated with metabolic and biosynthetic processes, including a few related to phosphorylation, as well as translation-associated functions and processes. At the beginning of the stationary growth phase, additional functions/processes start to be visible, including those related to ion/cation channel activities and ATP-related processes, as well as cell motility and carbohydrate metabolic processes. At the late stationary growth phase, multiple parallel, unconnected functions and processes, like DNA repair, transferases, and catalytic activities, were enriched, as were nucleotide biosynthetic processes that were already visible at the earlier time points. Of note, only the top 30 terms are shown in these maps; i.e., the functions and processes that are visible on top in the early stages likely are still active at the late stage, but they do not appear in the top 30.

### Phosphorylation related to annotated kinases.

We used the UniProt annotation of the C. difficile 630 genome (search with “cd630+kinase” in June 2019, 182 hits) to identify potential kinases and evaluate the auto-phosphorylation or phosphorylation of substrates of these kinases on the basis of findings in other organisms. Here, we focus on the putative protein kinases that are expected to phosphorylate serine, threonine, and tyrosine residues: SpoIIAB (CD630_07710) (32), RsbW (CD630_00100) (31), HPr kinase (CD630_34090) (30), PrkC/Stk (CD630_25780) (33), and CD630_21480. Other kinases identified by their annotation phosphorylate small molecules (nucleotides, metabolites) or are part of two-component regulatory systems and fall outside the scope of the present study.

**FIG 3:**
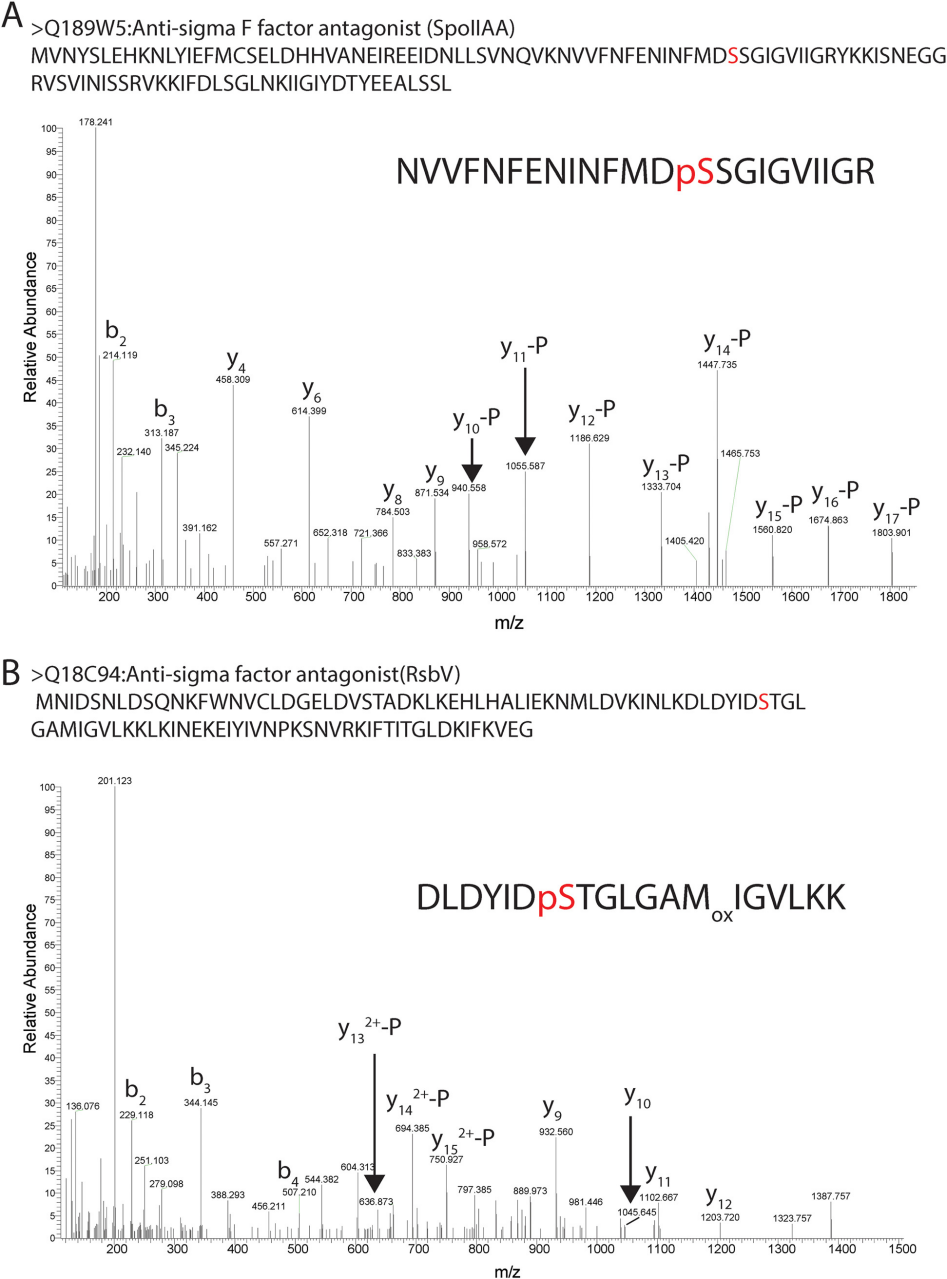
Spectra of conserved phosphorylation in regulatory proteins of C. difficile. (A) MS/MS spectrum of the tryptic phosphopeptide NVVFNFENINFMD**pS**SGIGVIIGR (**pS**, phosphoserine) from SpoIIAA, demonstrating phosphorylation of Ser-14 (Ser-56 in the full-length protein, indicated in red). (B) MS/MS spectrum of the tryptic phosphopeptide DLDYID**pS**TGLGAMIGVLKK (**pS**, phosphoserine) from RsbV, demonstrating phosphorylation of Ser-7 (Ser-57 in the full-length protein, indicated in red). Ions with “-P” have lost H_3_PO_4_.

SpoIIAB is an anti-sigma factor which in other bacteria is known to phosphorylate the anti-sigma factor F antagonist SpoIIAA during the process of sporulation (26, 32). In line with a role during sporulation, SpoIIAA phosphopeptides were exclusively identified in the 24-h sample, but not in samples taken at the mid-exponential or early stationary growth phase. The phosphorylation of SpoIIAA was found on Ser56 (LP = 0.83), Ser57 (LP = 0.55), Ser77 (LP = 1.00), Ser82 (LP = 0.84), and Ser83 (LP = 0.97) (Table S1). Of these, Ser56 corresponds to the equivalent serine of B. subtilis SpoIIAA (Ser58), which is known to be phosphorylated by SpoIIAB in that organism (37). The corresponding peptide was found in all C. difficile cultures after 24 h. Manual inspection of the tandem mass spectrometry (MS/MS) spectrum (Fig. 3A) confirmed the phosphorylation of Ser56 (corresponding to Ser14 in the tryptic peptide), because we observed neutral loss of phosphoric acid starting from the y_10_.

We also found phosphorylation of the kinase SpoIIAB itself (Table S1) at Ser13 (LP = 0.98), Ser17 (LP = 1.0), and Ser106 (LP = 1.00). Lower-confidence sites in SpoIIAB include Ser123 (LP = 0.50), Ser124 (LP = 0.49), and Thr111 (LP = 0.62). Of all these sites, only Ser106 was found consistently in all three 24-h cultures, and the assignment of the site was confirmed by manual inspection of the MS/MS data (Fig. S3A).

10.1128/msphere.00911-21.3 FIG S3 Spectra of conserved phosphorylation in regulatory proteins of C. difficile. (A) MS/MS spectrum of the tryptic phosphopeptide AMEPLYTSKPELDRpSGMGFTVMK from SpoIIAB, demonstrating the phosphorylation of Ser-15 (Ser-106 in the full-length protein, indicated in red). (B) MS/MS spectrum of the tryptic phosphopeptide NApSGLHARPAGMFVK from HPr, demonstrating the phosphorylation of Ser-3 (Ser-11 in the full-length protein, indicated in red). pS, phosphoserine. Ions with “-P” have lost H_3_PO_4_ FIG S3, PDF file, 0.5 MB. Copyright © 2022 Smits et al. 2022 Smits et al. https://creativecommons.org/licenses/by/4.0/ This content is distributed under the terms of the Creative Commons Attribution 4.0 International license.

RsbW is the anti-sigma factor for σ^B^, an important regulator of the stress response in Gram-positive bacteria (27, 31). The interaction of RsbW with σ^B^ is inhibited through the binding of the anti-σ factor antagonist RsbV to RsbW (27, 31). The interaction between RsbV and RsbW is negatively regulated by the RsbW-dependent phosphorylation of RsbV (27). We found two high-LP phosphopeptides for RsbV, indicative of phosphorylation of Ser57 (LP = 0.93) and Ser84 (LP = 1.00), and low-LP phosphorylation of Thr58 (LP = 0.56). Work with B. subtilis has identified three phosphorylation events, and two of these (Ser 56 and Thr57) are on the residues equivalent to the ones identified here in C. difficile RsbV (15). On the basis of manual inspection of the MS/MS data, we provide strong evidence for the phosphorylation of Ser57 in C. difficile RsbV (Fig. 3B). We also identified a significant number of phosphopeptides derived from the kinase RsbW itself, in most cultures, at the 24-h time point (Table S1): Ser87 (LP = 0.87), Ser89 (LP = 0.83), Thr90 (LP = 0.79), and Ser 99 (LP = 1.00).

HPr kinase (HprK/ScoC) can phosphorylate the phosphocarrier protein HPr (PtsH), a protein involved in the import of monosaccharides as part of the phosphotransferase system (PTS) (5). In B. subtilis, HPr kinase-mediated phosphorylation of HPr occurs on Ser46 (38, 39). We found the phosphorylation of the homologous residue (Ser45; LP = 1.00) of C. difficile HPr in all samples and additionally identified the high-confidence phosphorylation of Ser11 (LP = 1.00) and lower-confidence phosphorylation of Ser30 (LP = 0.85) and Thr31 (LP = 0.82) (Table S1). The equivalent site of Ser11 in B. subtilis HPr (Ser12) was also identified in a phosphoproteomics experiment (15). Importantly, the corresponding tryptic peptide of C. difficile HPr also contains a conserved histidine (His14), which is known to be phosphorylated through the phosphotransferase activity of the PTS (5, 40). The MS/MS spectrum of the tryptic peptide can be explained only by the phosphorylation of Ser2 in this peptide (Ser12 in the protein), not only on the basis of the absence of the pH of the immonium ion but, more importantly, also by the presence of the observed b and y ions (Fig. S3B). However, we cannot exclude the possibility that His14 of the HPr protein might also be phosphorylated. We found a single phosphopeptide derived from the HprK kinase itself; Thr299 (LP = 1.00) appears to be phosphorylated primarily in stationary growth phase, with most peptides identified after 24 h.

Whereas phosphorylation of Hanks-type serine-threonine kinases in other organisms has been described, this is not the case for the C. difficile proteins PrkC (33) and CD630_21480. Similarly, the substrates of the C. difficile kinases are virtually uncharacterized. We did not identify phosphopeptides derived from CD630_2148, but we found extensive phosphorylation of PrkC in the region between Ala158 and Lys183 (tryptic peptide AV**S**N**S**TMTNIGSIIGSVHYFSPEQAK, whose LP is >0.95 [underlined] and 0.70 < LP < 0.95 [bold]) throughout growth. Notably, the conserved residues Thr163 and Thr165 were also found to be phosphorylated in B. subtilis (41). Thr290 of B. subtilis PrkC, another residue that was identified in multiple studies to be phosphorylated (15, 41), is not conserved in C. difficile. However, two residues in the equivalent region of C. difficile PrkC (Thr287 and Thr302, both with an LP of 1.00) were identified, suggesting that phosphorylation in this region is structurally conserved. We also found phosphopeptides that indicate phosphorylation close to the N terminus of PrkC (Thr4 and Thr35; LP = 1.00) (Table S1).

A single substrate for PrkC has been characterized: CwlA (CD630_11350) (34). For this protein, phosphorylation was reported on Ser136 and Thr405, with the latter being specific for PrkC. In our analyses, we found multiple Ser-phosphorylated peptides, including Ser136 (LP = 1.00) and Thr405 (LP = 1.00) (Table S1). Additionally, it appears that PrkC is involved in cell wall homeostasis and antimicrobial resistance based on phenotypes of a *prkC* knockout strain (33). The authors postulate that part of the effects may be mediated by CD630_12830, a homolog of IreB of Enterococcus faecalis (33), though subsequent work indicates a major role for CwlA (34). In E. faecalis, IreB negatively controls cephalosporin resistance and is phosphorylated by the serine/threonine kinase IreK on a conserved N-terminal threonine residue (42). We confirmed phosphorylation of the equivalent residue (Thr8; LP = 1.00) across all time points in all samples (Table S1).

### Dynamic phosphorylation within a protein.

PrkC may also regulate cell division, such as DivIVA (33, 43–46). We identified high-confidence phosphopeptides derived from C. difficile DivIVA (CD630_26190), indicating phosphorylation of Ser75 (LP = 1.00), Ser82 (LP = 1.00), and Thr160 (LP = 0.98). The phosphorylation of DivIVA is one of the few examples of consistent phosphorylation at a specific site (Thr160) at early time points, but not after 24 h of culturing (Table S1). Interestingly, the cell division protein SepF (CD630_26220), which is encoded in the same gene cluster as *divIVA*, shows the same pattern of phosphorylation for Thr56 (LP = 0.96). In contrast, the other sites in DivIVA (Ser75 and Ser82) were found to be consistently phosphorylated in stationary growth phase but never at the mid-exponential phase. It therefore appears that the patterns of phosphorylation of DivIVA differ throughout growth. Exponential-growth-phase phosphorylation is not observed for all cell division proteins; for instance, ZapA (CD630_07910) and FtsZ (CD630_26460) are predominantly phosphorylated in stationary growth phase (Table S1). FtsZ is also phosphorylated in other bacteria (47–49).

We were interested to see if more proteins demonstrate dynamic phosphorylation. Manual inspection of the list of phosphopeptides identified two hypothetical proteins that show different patterns of phosphorylation in exponential phase from those in stationary growth phase, particularly with CD630_27170 and CD630_28470 (Table S1). Phosphorylation of CD630_27170 occurs exclusively on Ser573 (LP = 1.00) in exponential growth phase, at Ser573 and Ser286 (LP = 1.00) at the onset of stationary growth phase, and at multiple other sites at 24 h. CD630_28470 is phosphorylated at Ser186 (LP = 1.00) in exponential growth phase and at the onset of stationary phase but not in late stationary phase (Table S1). Together, these examples indicate that posttranslational modification within a particular protein in C. difficile can change in a growth phase-dependent manner.

### Indirect detection of cysteine phosphorylation.

In addition to using protein kinase-dependent phosphorylation, in which the phosphate group is donated by ATP, bacteria can use the phosphate group from phosphoenolpyruvate (PEP) in a process catalyzed by a PTS. This is of particular relevance for the import of monosaccharides into the cell, during which the sugars are converted to phospho-sugars (5). The initial enzymes involved in this cascade of events (enzyme I [PtsI] and Hpr [PtsH]) are phosphorylated at conserved histidine residues and do not have specificity for the different monosaccharides. The subunits of the enzyme II complex (EIIA, EIIB, and EIIC) provide the necessary specificity for different monosaccharides. In our phosphoproteomic data set, we found that many of these subunits of the different EII complexes are phosphorylated at serine and threonine residues (Table S1). Activation of the monosaccharide during uptake, however, requires the transfer of phosphate from a conserved cysteine residue of the EIIA and EIIB subunits, respectively. We accidently put carbamidomethylation of Cys as a variable, instead of as a fixed, modification during one of our database searches and serendipitously observed cysteine-containing peptides from EII complex subunits that did not appear to be phosphorylated (as expected based on our enrichment strategy) or carbamidomethylated (as expected from our proteomics workflow). Of note, when we searched data from a HeLa tryptic digest, which we use as a standard for our liquid chromatography (LC)-MS/MS setup, with the same parameters, we found very few free cysteines (18 out of 2,753 peptide spectrum matches of peptides containing a cysteine).

As an example of a tryptic peptide with a free cysteine in our C. difficile data, we observed the tryptic peptide ILVA**C**GAGIATSTIV**C**DRVER from the PTS system IIB component (CD630_10830, aa 4 to 24) containing two cysteines (in bold and underlined). In total, more than 300 peptide spectral matches for this peptide were identified in all our LC-MS/MS analyses of C. difficile phospho-samples. In all these identifications, the second cysteine (aa 16 in the tryptic peptide) was found to be carbamidomethylated, while the first cysteine at position 5 was a free cysteine (Fig. S4). Importantly, the first cysteine corresponds to the active-site cysteine that is transiently phosphorylated in the P-loop of EII subunits (50). We therefore hypothesize that the identification of such a peptide is due to the fact that these cysteines were phosphorylated during the initial steps of our workflow. This would allow for enrichment during the IMAC procedure and, if the phosphorylation persists during the reduction and alkylation steps, preclude carbamidomethylation. As no phosphorylated cysteine modifications were found in the LC/MS-MS, it appears that the phosphate group is lost during the later stages of our workflow (due to the instability of the phosphorylated cysteine under our experimental conditions).

10.1128/msphere.00911-21.4 FIG S4 Indirect identification of cysteine phosphorylation. MS/MS spectrum of the tryptic peptide LVACGAGIATSTIVCcamDRVER from the phosphotransfer system (PTS) component IIB (CD630_10830), in which the first cysteine (underlined) is a free cysteine, while the second is carbamidomethylated (cam). Download FIG S4, PDF file, 0.4 MB. Copyright © 2022 Smits et al. 2022 Smits et al. https://creativecommons.org/licenses/by/4.0/ This content is distributed under the terms of the Creative Commons Attribution 4.0 International license.

In conclusion, it appears that a one-step Fe-IMAC enrichment procedure can indirectly detect at least a subset of cysteine-phosphorylated proteins.

## DISCUSSION

In this study, we characterized the phosphoproteome of a laboratory strain of C. difficile at three different time points. We show that many proteins of C. difficile are phosphorylated in stationary growth phase, and an analysis of a subset of the identified phosphoproteins indicates that at least some of the phosphorylation sites are conserved between phylogenetically distinct organisms.

Many different methods have been used to analyze bacterial phosphoproteomes (1, 2, 11). A crucial step in these analyses is the method used to enrich for phosphopeptides (2, 4). In our study, we employed Fe-IMAC, as this has recently been shown to be a very effective way for analyzing bacterial phosphoproteomes (35, 36). The number of phosphoproteins identified here is in line with these studies and indicates that this is a suitable method for the analysis of phosphoproteins in C. difficile. We identified Ser, Thr, and Tyr phosphorylation and provided indirect evidence for Cys phosphorylation (Fig. 1A; see Table S1 in the supplemental material). Our workflow does not allow the identification of His phosphorylation, but recent studies have shown that the Fe-IMAC method can be adapted to detect the thermodynamically less stable His phosphorylation for future experiments (51, 52). The identification of other forms of phosphorylation, such as Arg phosphorylation, requires different approaches (53). We also observed multiply phosphorylated proteins (Table S1), as has been seen in other organisms as well (12, 54–56).

Our work provides a starting point for experimental validation of the identified phosphoproteins, as well as a dissection of the contribution of individual protein kinases and phosphatases. Generally, the actions of protein kinases are better understood than those of phosphatases (16). We identified site-specific phosphorylation in the protein kinase PrkC (Table S1). A knockout of the gene encoding this kinase demonstrates pleiotropic effects (33), and comparing wild-type and *prkC*-negative cells using our approach may help to elucidate the substrates of this protein.

The identified phosphopeptides also offer a possibility of characterizing the effect of phosphorylation on substrate proteins, by mutating residues in these proteins to nonphosphorylatable analogs (alanine for serine/threonine and phenylalanine for tyrosine) or phosphomimetic negatively charged amino acids (aspartate and glutamate) (2, 10). Such an approach may be preferable to mutating the kinases and phosphatases, as these can have overlapping specificities (57). However, mutating specific phosphorylation sites can lead to phosphorylation on neighboring sites, due to which the phenotypic consequences of the mutation may be only partially penetrant.

In previous phosphoproteomic analyses, a large divergence was often observed in the lists of phosphorylated proteins even within a single genus or species (2, 58, 59). In our experiments, we observed apparently conserved phosphorylation events on several proteins, including SpoIIA, SpoIIAB, RsbV, and others (Fig. 3; Table S1), which may appear in contrast to these observations. Part of the reason for the limited overlap in previous experiments may have been the diversity in enrichment methods and experimental protocols, limited sensitivities of the workflows, and variability in sampling methods and time points. Our workflow allows the reliable identification of a large set of phosphoproteins in comparison with those found by other studies (11, 12, 16, 17). Moreover, we applied rigorous quality control of phosphosite assignment. It is likely that developments in mass spectrometry instrumentation, data acquisition, and analysis pipelines will reveal that many more processes in fact may be regulated by conserved phosphorylation events. For example, a recent phosphoproteomic analysis of Staphylococcus aureus increased the number of phosphosite identifications over 20-fold compared to those of earlier analyses, thereby also increasing the number of processes affected (35). Cross-species comparisons may be facilitated by dedicated databases of phosphorylated proteins, such as PHOSIDA and dbPSP 2.0 (60, 61), and ultimately lead to improved predictions of phosphorylation in bacteria (62).

Our work indicates that levels of protein phosphorylation in C. difficile may differ both spatially (within a protein) and temporally (between time points). Specifically, we observe low levels of phosphorylation in exponential growth phase, whereas phosphorylation increases markedly upon entry into stationary growth phase. Of note, we observed a highly variable number of phosphoproteins at the entry into logarithmic growth phase, with two out of three samples showing clearly elevated levels, whereas a single biological replicate resembled the profile observed in exponential growth phase (Fig. 1B). This suggests that the phosphorylation state may change rapidly and supports the notion that the sampling time point may explain differences between phosphoproteomic data sets. We also report that specific phosphorylation patterns can be observed within a protein, as exemplified by DivIVA and SepF (Table S1). This has also been observed for other organisms; for instance, in Streptomyces coelicolor, 58/85 phosphorylation sites were differentially phosphorylated during differentiation (13). Interestingly, Thr160 of C. difficile DivIVA is located in the putative tetramerization region of the protein (63), and phosphorylation at the C terminus of DivIVA has also been observed for S. pneumoniae, where is it mediated by the eSTK protein StkP (46). Mutations of this region lead to aberrant cell morphology, suggesting that phosphorylation of Thr160 in C. difficile might have functional consequences. Thr56 of C. difficile SepF is directly adjacent to the predicted compact domain that mediates self-interaction and interaction with the cell division protein FtsZ (64). The functional consequences of the dynamic phosphorylation of DivIVA and SepF remain to be elucidated.

We focused our in-depth analyses on those proteins for which the phosphosite assignment could be done with high probability; where necessary, we manually verified the automatic identifications. Nevertheless, we also observed peptides for which it was not possible to assign the modification to a particular residue, similar to what others did (59). For those proteins, alternative fragmentation techniques (65) or processing of the sample—using, for instance, proteases other than trypsin—might yield better results.

The phosphoproteome described here complements existing omics approaches for C. difficile, such as genomics, transcriptomics, proteomics, and metabolomics. Integration of these data may lead to a systems-level understanding of C. difficile physiology and, more broadly, may contribute to our understanding of the role of phosphorylation in the regulation of bacterial pathogenesis (16).

## MATERIALS AND METHODS

### Chemicals.

Unless noted otherwise, chemicals were obtained from Sigma-Aldrich Chemie.

### Cell culture.

C. difficile 630Δ*erm* cells (66, 67) were grown at 37°C in brain heart infusion medium (BHI; Oxoid) supplemented with yeast extract (YE) in a Don Whitley VA-1000 workstation (10% CO_2_, 10% H_2_, and 80% N_2_ atmosphere). An initial preculture was grown until an optical density at 600 nm (OD_600_) of 0.36. Three independent cultures were started by adding 31 mL of the preculture to 400 mL fresh BHI-YE for each new culture. Samples were taken at the mid-exponential (3.25 h postinoculation), early stationary (5.25 h postinoculation), and late stationary (24 h postinoculation) phases (see Fig. S1 in the supplemental material). To get roughly equivalent amounts of cells, 100 mL was taken at the mid-exponential time point (OD_600_, 0.96), while 50 mL was taken at the two later time points (OD_600_, ∼2).

JY cells were grown at 37°C in Iscove's modified Dulbecco's medium supplemented with 10% heat-inactivated fetal bovine serum and l-glutamine. Both eukaryotic and bacterial cells were harvested by centrifugation (3,184 × *g*, 10 min) and washed three times with 25 mL of phosphate-buffered saline (PBS; Fresenius Kabi Nederland BV). Cell pellets were subsequently stored at −20°C until further use.

### Sample preparation.

Cell pellets were resuspended in 3 mL lysis buffer [8 M urea–50 mM Tris-HCl, pH 7.4, 1 mM orthovanadate, 5 mM Tris(2-carboxyethyl)phosphine (TCEP), 30 mM chloroacetamide (CAA), PhosSTOP phosphatase inhibitor (Thermo Fischer Scientific), cOmplete mini EDTA-free protease inhibitor, 1 mM MgCl_2_] and incubated for 20 min at room temperature. Cells were lysed by sonication (Soniprep 150 ultrasonic disintegrator [MSE]; 5 × 30 s; amplitude, 12 μm). In between sonication steps, samples were cooled on ice for 30 s. Next, samples were centrifuged for 15 min at 7,200 × *g* at 4°C. The urea concentration was adjusted to 6 M by the addition of 1 mL of 50 mM Tris-HCl, pH 7.4, after which 1 μL Benzonase (250 U/μL) was added and samples were incubated for 2 h at room temperature.

Proteins were precipitated by first adding 16 mL of methanol (Actu-All Chemicals) and mixing, followed by the addition of 4 mL of chloroform (Merck Millipore) and mixing. After the addition of 12 mL of Milli-Q water (obtained from an Elga Pure Lab Chorus 1 machine), the samples were mixed by vortexing. Samples were then centrifuged for 15 min at 11,000 × *g*, followed by another 5 min at 11,000 × *g* (this resulted in better separation of the two phases than one round of 20 min of centrifugation). The protein precipitates at the interphase were collected and washed twice with 2 mL methanol, followed by 5 min of centrifugation at 11,000 × *g*. The protein pellets were air-dried and resuspended in 5 mL 25 mM NH_4_HCO_3_, pH 8.4.

Trypsin was added at a ratio of 1:25 (wt/wt), and overnight digestions were performed at 37°C. The next day, samples were centrifuged for 10 min at 11,000 × *g*, and the supernatants were collected. Desalting of the samples was performed using Oasis HLB 1-mL Vac cartridges (Waters). Briefly, the cartridge was washed once with 1 mL acetonitrile (Actu-All Chemicals)-H_2_O (90/10 [vol/vol]), equilibrated with Milli-Q H_2_O-acetonitrile-formic acid (Fluka Analytical) (95/3/0.1 [vol/vol/vol]) (solution A, 2 × 1 mL). Following sample loading, the column was washed with solution A (3 × 1 mL), after which peptides were eluted with acetonitrile-water-formic acid (30/70/0.1 [vol/vol/vol]). Tryptic peptides were lyophilized (Salm en Kipp Christ RVC 2 to 18 CD plus) and stored at −20°C until use.

### Phosphopeptide enrichment.

A one-step phosphopeptide IMAC enrichment procedure was performed using a 4- by 50-mm ProPac IMAC-10 analytical column (Thermo Fisher Scientific). For the JY cells, an equivalent of 5 mg of protein was used for one IMAC purification, while for the C. difficile cells, the total amount of peptides from each sample was applied, with an average of 11 (±1.4 standard deviations [SD]) mg protein per sample. Prior to each IMAC run, the column was stripped using 50 mM EDTA-0.5 M NaCl, pH 4, and charged with 25 mM FeCl_3_ in 100 mM acetic acid, according to the manufacturer’s instructions, using an offline pump system (Shimadzu). After this, the column was connected to an Agilent 1200 chromatography system running at a flow rate of 0.3 mL/min. Prior to sample loading, the column was equilibrated in solvent A (water-acetonitrile-trifluoroacetic acid 70/30/0.07 [vol/vol/vol]) for 30 min at a constant flow rate of 0.3 ml/min.

Tryptic peptides (resuspended in 260 μL solvent A, of which 250 μL was injected) were loaded, and nonphosphopeptides were removed by washing the column for 20 min with solvent A. Peptide separation was performed using a linear gradient from 0% to 45% of solvent B (0.5% [vol/vol] NH_4_OH). The complete peak fraction of phosphopeptides between 46 and 49 min was manually collected and lyophilized prior to mass spectrometric analysis.

### LC-MS/MS analysis.

Lyophilized peptides were reconstituted in 100 μL water-formic acid (100/0.1 [vol/vol]) and analyzed by on-line C_18_ nano-high-performance liquid chromatography (nano-HPLC) MS/MS with a system consisting of an Easy nLC 1200 gradient high-performance liquid chromatography (HPLC) system (Thermo Fisher Scientific, Bremen, Germany) and an Orbitrap Fusion Lumos Tribrid mass spectrometer (Thermo Scientific). Samples (10 μL, in duplicate) were injected onto a homemade precolumn (100 μm by 15 mm; Reprosil-Pur C_18_-AQ, 3 μm; A. Maisch, Ammerbuch, Germany) and eluted on a homemade analytical nano-HPLC column (30 cm by 75 μm; Reprosil-Pur C_18_-AQ, 3 μm). The gradient was run from 2% to 36% solvent B (water-acetonitrile-formic acid [20/80/0.1, vol/vol/vol]) in 120 min. The nano-HPLC column was drawn to a tip of ∼5 μm, which acted as the electrospray needle of the MS source.

The Lumos mass spectrometer was operated in data-dependent MS/MS mode for a cycle time of 3 s, with the higher-energy C-trap dissociation (HCD) collision energy at 32 V and recording of the master scan 2 (MS2) spectrum in the Orbitrap. In MS1, the resolution was 120,000 and the scan range *m/z* was 300 to 1,500 at an AGC target of 400,000 at a maximum fill time of 50 ms. Dynamic exclusion was set after 1, with an exclusion duration of 60 s. Charge states 2 to 4 were included. For MS2, precursors were isolated with the quadrupole, with an isolation width of 1.2 Da. First mass was set to 110 Da. The MS2 scan resolution was 30,000, with an AGC target of 50,000 at a maximum fill time of 60 ms.

### Data analysis.

MaxQuant software (version 1.5.1.2) was used to process the raw data files, which were searched against the C. difficile strain 630 database (4,103 entries) or human canonical database (67,911 entries). The mass tolerances for MS1 and MS2 were set to 4.5 and 20 ppm, respectively. Trypsin was selected as the enzyme with a maximum of two missed cleavages. Carbamidomethylation of cysteines was selected as a fixed modification and phosphorylation on serine, threonine, and tyrosine; in addition, oxidation of methionine and acetylation of the protein N terminus were selected as variable modifications. The match-between-runs option was selected with a window of 2 min. The false-discovery rate at the peptide level was set to 1%, and for modified peptides, a score cutoff of 40 was used.

The MaxQuant output table “phospho (STY)Sites.txt” (Table S1) was used for further analysis of the phosphopeptides. For the stringent phosphorylation site assignment, only peptides with a localization probability score of >0.95 were selected. Because we used the match-between-runs option, we only used unique peptides without considering the phosphorylation site (i.e., not considering phospho-isomers) for comparison of different samples.

Gene ontology protein/gene set enrichment analysis was performed using a sorted list of all identified phosphoproteins at each analysis time point, i.e., mid-exponential, beginning stationary, and late stationary growth phases. The sorting was based on the abundance of the phosphoproteins identified and determined by the ion intensity, as reported by the mass spectrometer in the output of MaxQuant (cutoff at a score of 40 and false-discovery rate at 1%). We considered the phosphoproteins that were reported at least once in any of the three replicates. Each protein identification was considered only once in the sorted list, with a rank determined by the most intense results that we have obtained in any of the three replicates by any protein-associated phosphopeptide. The enrichment was performed using the weighted Kolmogorov-Smirnov-like statistic as implemented in the common gene set enrichment analysis. We limited our analysis to the top 30 enriched gene ontology terms. We generated enrichment maps that represent the relationship between the enriched terms as a network. The edges of an enrichment map correspond to the number of shared proteins between the associated terms. These maps give a higher-level overview of the enrichment analysis and allow identifying functional modules with ontology terms that are related to each other by the underlying protein set used. For the functional analysis, we used all gene ontology annotations of C. difficile as reported by the Gene Ontology database hosted by EBI (as of 15 August 2021). For the functional analysis and visualization, we used R 3.6.2 and the Bioconductor library Cluster Profiler.

### Data availability.

All data are contained within the manuscript or the associated supplemental material or are available from the authors upon request. The mass spectrometry proteomics data have been deposited into the ProteomeXchange Consortium via the PRIDE partner repository (68) with the data set identifier PXD029475.
