# Supplementary figures and images for: Clostridioides difficile Phosphoproteomics Shows an Expansion of Phosphorylated Proteins in Stationary Growth Phase
Source: mSphere. 2022 Jan 5;7(1):e00911-21. doi: 10.1128/msphere.00911-21 (PMC8730811; doi:10.1128/msphere.00911-21)

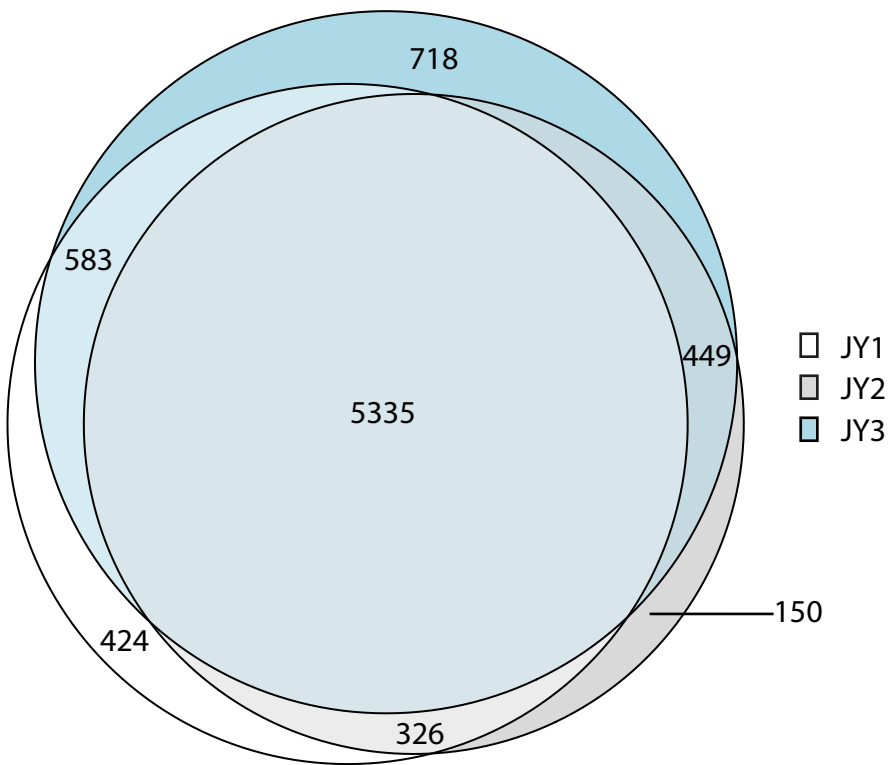

Supplemental Figure 2

Supplement: FIG S2 [file msphere.00911-21-sf002.pdf]

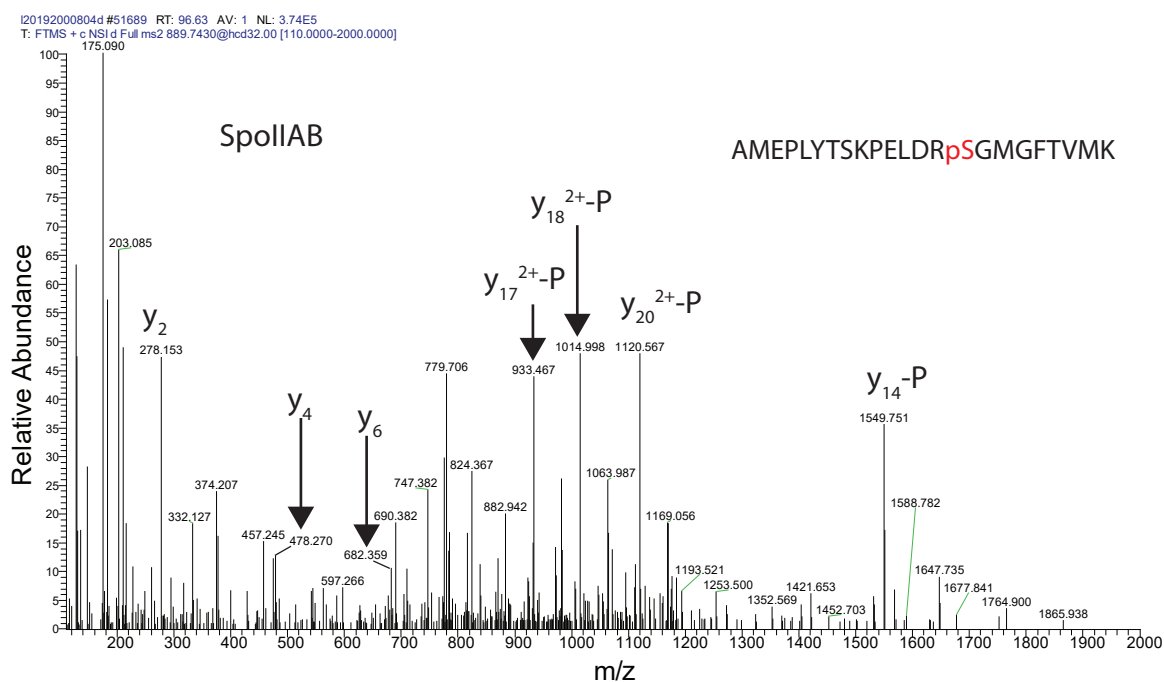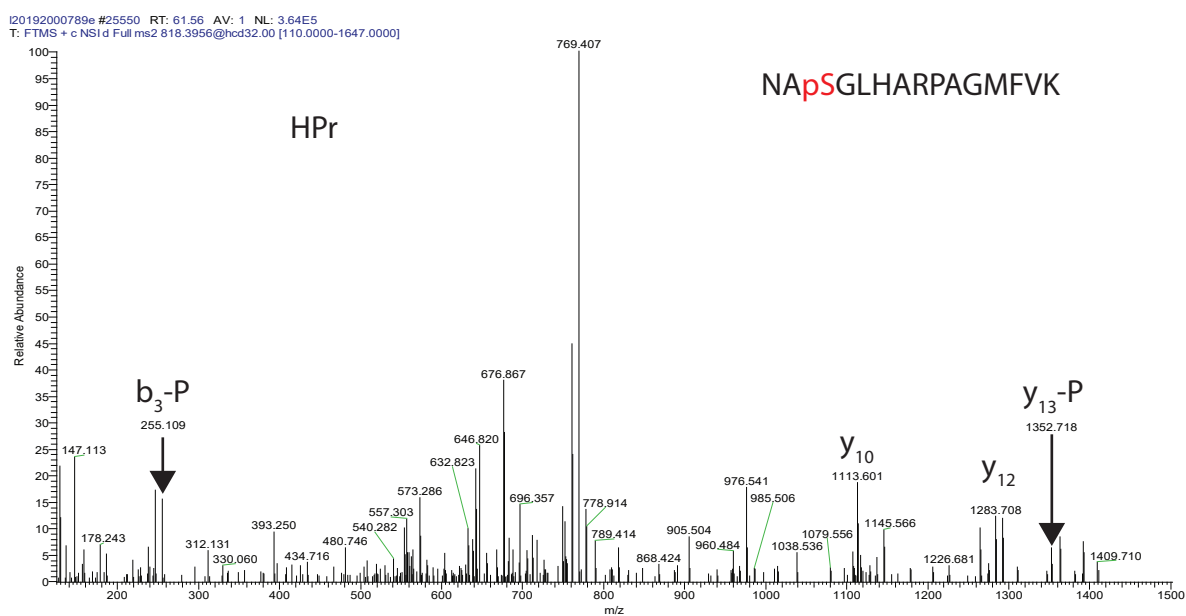

Supplemental figure 3

Supplement: FIG S3 [file msphere.00911-21-sf003.pdf]

L20192000804e #76178 RT: 131.81 AV: 1 NL: 1.86E5  
T: FTMS + c NSI d Full ms2 735.7236@hcd32.00 [110.0000-2000.0000]

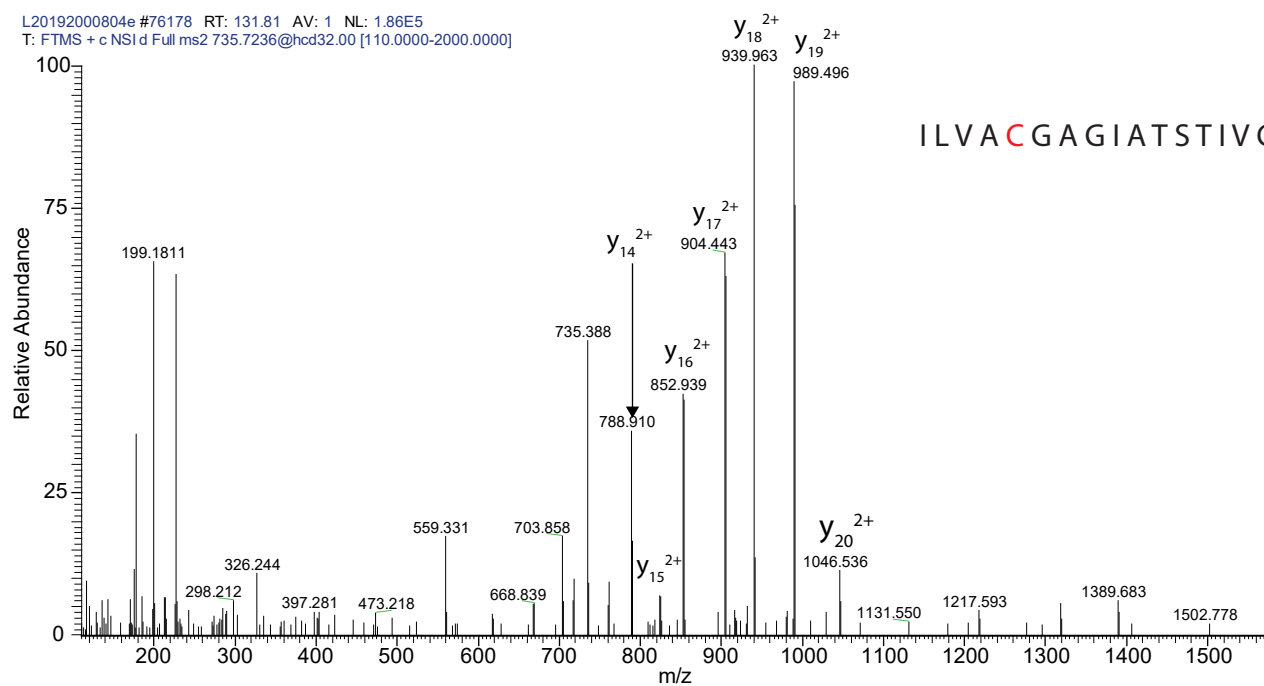

ILVACGAGIATSTIVC<sub>cam</sub> DRIVER

Supplemental figure 4

Supplement: FIG S4 [file msphere.00911-21-sf004.pdf]
