# Supplementary material for: Clostridioides difficile Phosphoproteomics Shows an Expansion of Phosphorylated Proteins in Stationary Growth Phase
Source: mSphere. 2022 Jan 5;7(1):e00911-21. doi: 10.1128/msphere.00911-21 (PMC8730811; doi:10.1128/msphere.00911-21)
Supplement: FIG S1 [file msphere.00911-21-sf001.pdf]

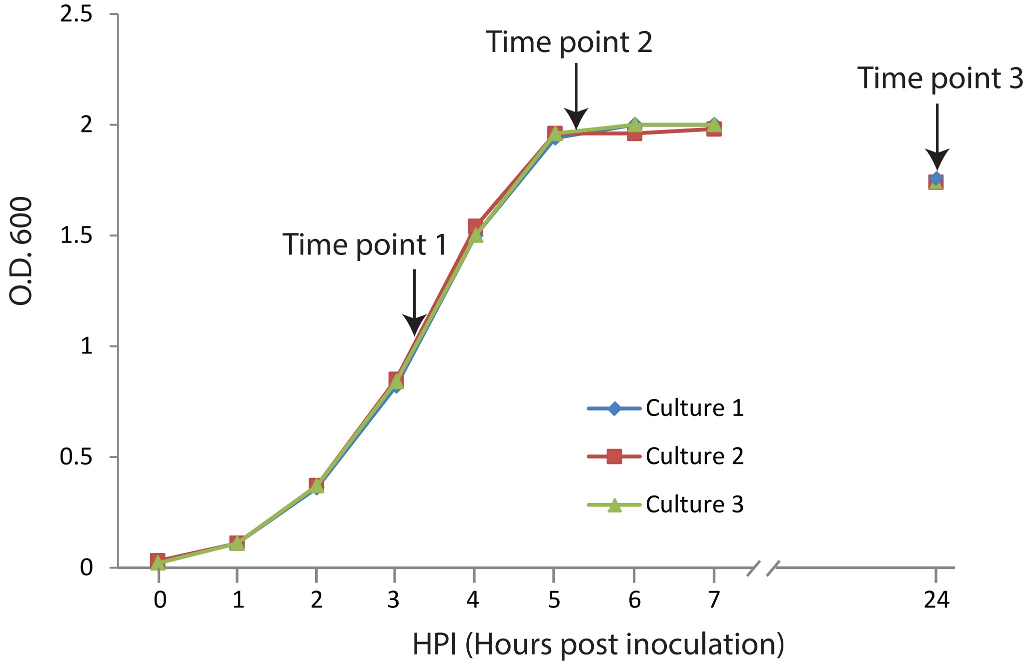

Time point 1: Mid-exponential phase (3h15 HPI, OD600=1.0)
Time point 2: Start stationary phase (5h15 HPI, OD600=2.0)
Time point 3: Late stationary phase/overnight (24h HPI, OD600=1.7 ± 0.07)

Total protein amount (mg):

	Culture 1	Culture 2	Culture 3
Time point 1	9.4	10.9	10.8
Time point 2	12.4	12.3	12.1
Time point 3	10.6	8.1	10.2

Supplemental Figure 1
